# Pain Relief Dependent on IL-17–CD4^+^ T Cell–β-Endorphin Axis in Rat Model of Brachial Plexus Root Avulsion After Electroacupuncture Therapy

**DOI:** 10.3389/fnins.2020.596780

**Published:** 2021-02-09

**Authors:** Zihang Xu, Yangzhuangzhuang Zhu, Jun Shen, Lin Su, Yifei Hou, Mingxi Liu, Xiaoning Jiao, Xiao Chen, Shiguo Zhu, Yechen Lu, Chao Yao, Lixin Wang, Chenyuan Gong, Zhenzhen Ma, Chunpu Zou, Jianguang Xu

**Affiliations:** ^1^School of Basic Medical Sciences, Shanghai University of Traditional Chinese Medicine, Shanghai, China; ^2^Department of Orthopedics, Guanghua Hospital of Integrative Chinese and Western Medicine, Shanghai, China; ^3^Arthritis Institute of Integrated Traditional Chinese and Western Medicine, Shanghai Academy of Traditional Chinese Medicine, Shanghai University of Traditional Chinese Medicine, Shanghai, China; ^4^School of Rehabilitation Science, Shanghai University of Traditional Chinese Medicine, Shanghai, China; ^5^Department of Hand Surgery, Huashan Hospital, Fudan University, Shanghai, China

**Keywords:** BPRA, β-endorphin, CD4^+^ T lymphocytes, IL-17, neuropathic pain

## Abstract

**Background and purpose:**

Neuropathic pain is the typical symptom of brachial plexus root avulsion (BPRA), and no effective therapy is currently available. Electroacupuncture (EA), as a complementary and alternative therapy, plays a critical role in the management of pain-associated diseases. In the present study, we aimed to reveal the peripheral immunological mechanism of EA in relieving the pain of BPRA through the IL-17–CD4^+^ T lymphocyte–β-endorphin axis.

**Methods:**

After receiving repeated EA treatment, the pain of BPRA in rats along with the expressions of a range of neurotransmitters, the contents of inflammatory cytokines, and the population of lymphocytes associated were investigated. CD4^+^ T lymphocytes were either isolated or depleted with anti-CD4 monoclonal antibody. The titers of IL-17A, interferon-γ (IFN-γ), and β-endorphin were examined. The markers of T lymphocytes, myeloid-derived suppressor cells (MDSCs), dendritic cells (DCs), macrophages, and natural killer (NK) cells were assessed. The activation of the nuclear transcription factor κB (NF-κB) signaling pathway was tested.

**Results:**

The pain of BPRA was significantly relieved, and the amount of CD4^+^ T lymphocytes was increased after EA treatment. The release of β-endorphin was up-regulated with the up-regulation of IL-17A in CD4^+^ T lymphocytes. The titer of IL-17A was enhanced, leading to an activated NF-κB signaling pathway. The release of β-endorphin and the analgesic effect were almost completely abolished when CD4^+^ T lymphocytes were depleted.

**Conclusion:**

We, for the first time, showed that the neuropathic pain caused by BPRA was effectively relieved by EA treatment via IL-17–CD4^+^ T lymphocyte–β-endorphin mediated peripheral analgesic effect, providing scientific support for EA clinical application.

## Introduction

As the most serious form of peripheral nerve injury, brachial plexus root avulsion (BPRA) results in the paralysis of ipsilateral upper limbs, and it is usually caused by high-energy trauma, such as traffic accidents and gunshots (Meng et al., 2019). BPRA-caused pain is largely featured by the quick onset of pain (an effect presenting right away following the injury) and the long-lasting progress of neuropathic pain, which is described as crushing, squeezing, or burning (Chen et al., 2019; Wang T. et al., 2019). Both patients and clinicians have found that the pain associated with this injury is often intolerable, which is worse than a non-functioning limb. Unfortunately, no effective treatments are currently available (Laohaprasitiporn et al., 2018).

In traditional Chinese medicine (TCM), the pain occurring after BPRA is related to flaccidity syndrome (Zhang et al., 2014). As a vital component of TCM, acupuncture has been practiced for thousands of years in Eastern countries, and it is usually used as an effective complementary and alternative therapy to relieve different types of pain (Eshkevari, 2017). As a modified approach of acupuncture, electroacupuncture (EA) employs electrical stimulation. Besides, many clinical trials have shown that acupuncture or EA stimulation also plays a critical role in the management and therapy of immune-related disorders, such as allergic disorders, infections, autoimmune diseases, and immunodeficiency syndromes (Wang et al., 2017; Lv et al., 2019). Nowadays, increasing evidence has shown that the analgesic effects of acupuncture are tightly associated with immune regulation. It is well known that acupuncture or EA promotes the secretion of certain neurotransmitters, especially opioids, in the central nervous system (Tjen et al., 2018). It has been shown that there is a positive correlation between the activation of immune cells and the contents of opioid peptides in the inflammatory environment. It is known that lymphocyte infiltration is increased during inflammation. The locally secreted opioid peptides bind to opioid receptors to generate analgesic effects, revealing that the number of infiltrating immune cells plays an important role in the peripheral analgesic action (Plein and Rittner, 2018; Wang Z. et al., 2019). Endogenous opioid peptide family mainly consists of enkephalin, β-endorphin, and dynorphin. Elevated circulating levels of β-endorphin, which mostly comes from hypophysis, are a potential biomarker for endogenous opioid analgesic capacity, and such elevation is often related to pain (Cravana et al., 2017). Leukocytes in certain circumstances can produce and secrete β-endorphin, offering the molecular mechanisms for communication with the neuroendocrine system (Basso et al., 2016).

Interleukin 17 (IL-17), as a proinflammatory cytokine, is primarily synthesized by a series of independent cluster of differentiation (CD)4^+^ T cell subsets, and it participates in a broad range of inflammatory diseases and recruitment of inflammatory cells to injured sites (Ruiz de Morales et al., 2020). The role of IL-17 in analgesia may be similar to that of T lymphocytes. However, IL-17 presents an analgesic effect under some conditions, while it also induces pain under other conditions, which may depend on the microenvironment of the specific disease in which IL-17 is located. For instance, studies have shown that the absence of IL-17 leads to reduced infiltration of immune cells to relieve the hypersensitivity of pain in a mouse model of partial sciatic nerve ligation (Day et al., 2014). However, on the other hand, the increased IL-17 level can promote the infiltration of CD4^+^ T lymphocytes, thus increasing the secretion of peripheral β-endorphin, which may also play a vital role in pain relief. Nevertheless, its underlying mechanism remains unknown. In our present study, we, for the first time, aim to investigate whether the pain of BPRA was significantly relieved by IL-17–CD4^+^ T cell–β-endorphin mediated analgesic effect after the model rats received repeated EA treatment. Moreover, the content of β-endorphin was elevated by increasing IL-17A (a member of the IL-17 ligands) in CD4^+^ T lymphocytes via activating the nuclear transcription factor κB (NF-κB) signaling pathway, and when CD4^+^ T lymphocytes or IL-17A was depleted, the expression of β-endorphin was almost entirely abolished.

## Materials and Methods

### Animals

Healthy male Sprague–Dawley rats (weight, 200–220 g; 8–10 weeks old) were purchased from Shanghai Slack Laboratory Animal Limited Liability Company (Shanghai, China), and animals were housed under a standard condition (12/12-h light–dark cycle, 20–22°C) and given free access to diet and water. The animals were kept for at least 1 week before operation or test. All the animal-related protocols and procedures were carried out according to the Guide for the Care and Use of Laboratory Animals described by the US National Institutes of Health. The animal study was reviewed and approved by the Ethics Committee of Shanghai University of Traditional Chinese Medicine.

### Animal Model

The BPRA surgery was performed on the right side of rats as previously described (Hou et al., 2020). Briefly, animals were intraperitoneally injected with sodium pentobarbital at a dose of 40 mg/kg, and then anesthetized rats were placed in a prostrate position on a sterile surgical station. Using T2 vertebrae as landmarks, an approximate 4-cm skin incision was made through the dorsal midline from the occipital bone to angulus superior scapulae. The brachial plexus roots were accessed using the posterior approach by dividing longissimus capitis muscle, semispinal muscle of the neck, biventer cervicis, and complex muscle under an operative microscope (magnification × 10). The muscles on the vertebral plate and spinal process were dissected, and the right nerve root and the brachial plexus roots were exposed from C5 to T1 via hemilaminectomy from C4 to T1. Once the roots were clearly defined, they were directly avulsed from the spinal cord. Hemostasis during and after surgery was achieved by bipolar electrocoagulation, and the surgical incisions were sprinkled with penicillin powder. Animal models were established by the same researcher. At 1 week after the surgery, the rats were stochastically assigned into two groups as follows: the EA group received EA treatment and the control group received no treatment.

### Assessment of Nociceptive Behavior

The behavior assessment was applied as described previously (Hou et al., 2020). In addition, both tests were applied on the contralateral (non-injured) side due to the observed autonomic behavior of biting the injured digits in model rats. Because the pain threshold of the contralateral side would be reduced, the sensitivity to pain was enhanced by the neuropathic pain aroused by brachial plexus avulsion of the ipsilateral side due to the injury-caused peripheral sensitization of nociceptors.

#### Electronic Von Frey Test

The rats were housed in the organic cages on a metal mesh for 30 min before testing. The withdrawal response was elicited by electronic von Frey filaments. The time was recorded when a withdrawal response was detected or when a cutoff value of 50 g was achieved. Each animal was measured five times with a 3-min resting period between tests.

#### Hargreaves Test

Animals were individually housed in an organic cage on a 3-mm-thick glass plate, followed by adaptation to the environment for 30 min. The BEAM value (irradiation intensity) was set to 5, and the heat stimulation was performed by applying a radiant heat source. The paw withdrawal latency in response to a heat stimulus was automatically determined, which was recorded as the time from onset of the stimulus to withdrawal response. The automatic cutting time was 25 s to prevent tissue damage. Each animal was measured five times with an interval of 3 min, and the average of five measurements was taken.

### Drugs and Reagents

Red blood cell (RBC) lysis kit was purchased from Biosharp, China. FITC anti-rat CD8a, PE anti-rat CD4, APC anti-rat CD3, anti-rat CD28, PerCP/Cy5.5 anti-rat CD11b/c, FITC anti-rat CD103 (αE Integrin), and FITC anti-rat CD335 NKp46 antibodies were obtained from Biolegend, United States. Anti-rat CD68 antibody was supplied by Bio-Rad, United States. Brefeldin A solution (1000×) was provided by eBioscience; Tissue RNA Purification Kit PLUS, 4× Reverse Transcription Master Mix, and 2× SYBR Green qPCR Master Mix were purchased from EZ Bioscience, China. Anti-rat β-endorphin antibody was obtained from Neuromics, United States. Anti-rat CD3 and anti-rat granulocyte (HIS48) antibodies were supplied by Abcam, United States. Anti-rat IL-17A antibodies were provided by Absin, China. MojoSort Rat Isolation Kit was purchased from STEMCELL, United States. Rat GADPH was selected as a housekeeping gene and synthesized by Sangon Biotech, China. Quick Detect β-endorphin (rat) enzyme-linked immunosorbent assay (ELISA) kit was obtained from Bio Vision, United States. Rat interferon-γ (IFN-γ) ELISA kit was purchased from BOSTER, China. Rat IL-17A was supplied by PEPROTECH, United States. Rat IL-17A ELISA kit was provided by Dakewe, China. Anti-CD4 antibody and control rat IgG were purchased from Bio X cell, United States. Anti-IL-17A antibody control mouse IgG was supplied by eBioscience^TM^, United States. Phosphatase inhibitor and protease inhibitor were purchased from Beyotime, China. Rabbit monoclonal phospho-IKKα/β, rabbit monoclonal IKKβ, rabbit monoclonal p65, rabbit monoclonal phospho-NF-κB p65, rabbit monoclonal IκBα, and rabbit monoclonal phospho-IκBα antibodies were obtained from Cell Signaling Technology, United States. Chemiluminescent HRP Antibody Detection Reagent was supplied by Millipore, United States.

### Acupuncture Therapy and *in vivo* Antibody Treatment in Rats

The EA treatment was carried out once a day for 2 months. The acupoint was located 0.5 cm beside the C5–C7 spinous process (Supplementary Figure S1). Before starting, the fur over the acupoint was shaved, followed by cleaning of the exposed skin. During the EA therapy, disposable intradermal acupunctures (0.30 × 13 mm, Hwato, China) were inserted into the acupoint at a depth of 3 mm. An SDZ-II nerve and muscle stimulator (Hwato, Suzhou Medical Appliance Factory, China) was used to perform the EA stimulation at an intensity of 1.5 mA, a frequency of 2/15 Hz, and a dilatational wave of 30 min. For the depletion of CD4^+^ T cells, rats were treated with 5 mg/kg anti-CD4 antibody, or control rat IgG by intraperitoneal injections twice a week for 2 months. For neutralization of IL-17A, rats were treated with 10 mg/kg anti-IL-17A antibody, or control mouse IgG by intraperitoneal injections once a week for 2 months.

### Tissue Processing and Flow Cytometry

All tissues were used as freshly prepared. After rats were anesthetized with 1% sodium pentobarbital, whole blood was collected from the abdominal aorta, and spleen and bone marrow were also harvested by mechanical dissociation, followed by RBC lysis. Suspensions were washed with phosphate-buffered saline (PBS) prior to cell surface staining with FITC anti-rat CD8a, PE anti-rat CD4, APC anti-rat CD3, Per CP/Cy5.5 anti-rat CD11b/c, FITC anti-rat CD103 (αE Integrin), PE anti-rat CD80, FITC anti-rat OX-62, FITC anti-rat CD335 NKp46, and rat anti-CD68 antibodies. Data were acquired and analyzed on a Cyto FLEX S using Cyto Expert software (Beckman Coulter).

### Reverse Transcription-Quantitative Polymerase Chain Reaction

Total RNA was isolated from the spleen using the Tissue RNA Purification Kit PLUS, which was then reversely transcribed into cDNA using 4× Reverse Transcription Master Mix. The primers used in the present study were as follows: HTR1A (forward 5′-GATCTCGCTCACTTGGCTCATTGG-3′; reverse 5′-GCTGTCCGTTCAGGCTCTTCTTG-3′), HTR2A (forward 5′-GCTGCCTGCTTGCCGATG AC-3′; reverse 5′-TCT CTGTGGATGGACCGTTGGAAG-3′), OPRK1 (forward 5′-GC TGGTGCTGGT AGTGGTTGC-3′; reverse 5′-GTGCTCTGG CGCTCCATTCG-3′), PDYN (forward 5′-CAGACTGCCT GTC CTTGTGTTCC-3′; reverse 5′-CTTGGTCAGTTCCGTGTAGC CTTC-3′), PENK (forward 5′-GGTCCTGCCTCCTGGCTAC AG-3′; reverse 5′-GCAAGGATCTCGCCTCCATTGG-3′), PO MC (forward 5′-AGGCGTGCGGAGGAAGAGAC-3′; reverse 5′-GCGTTCTTGATGATGGCGTTCTTG-3′), MOR (forward 5′-GCGGTCTGCCACCCTGTC-3′; reverse 5′-CACGAAGGCG AAGAGGAACAC-3′), DRD1(forward 5′-GCGTCCATTCTGA ACCTCTG-3′; reverse 5′-CGTCCTGCTCAACCTTGTG-3), DR D2 (forward 5′-GGTCTACTCCTCCATTGTCTCA-3′; reverse 5′-CATCCATTCTCCGCCTGTT C-3′), and α2-AR (forward 5′-GTGTGCTTGTTTCTGTCTTG-3′; reverse 5′-TATCGGGT AGGTTTCTTCC A-3′). GADPH was employed as a housekeeping gene. Reverse transcription-quantitative polymerase chain reaction (RT-qPCR) was performed on a Quanti-Studio 3 (ThermoFisher) PCR instrument using the 2× SYBR Green qPCR Master Mix kit. The relative gene expression was calculated using the 2^ΔΔ*Ct*^ method.

### Cytokine Array

The serum from peripheral blood was collected under different treatment conditions. The assay was performed using a rat 6-plex suspension cytokine array to compare the concentrations of IL-12, IL-12, IL-13, IL-17A, eotaxin, Granulocyte colony stimulating factor (G-CSF), Granulocyte-macrophage colony stimulating factor (GM-CSF), IFN-γ, Keratinocytechemoattractant (KC), monocyte chemotactic protein-1 (MCP-1), Macrophage-inflammatory protein-1α (MIP-1α), MIP-1β, and tumor necrosis factor-α between the two groups. A standard curve was generated following the manufacturer’s instructions, and all the obtained values were within the effective detection limits.

### Western Blotting Analysis

Spleen lysates were obtained using RIPA lysis buffer supplemented with phosphatase inhibitor and protease inhibitor, and the obtained tissue lysates were denatured at 99°C for 15 min. Equal amounts of proteins (40 μg) were subjected to sodium dodecyl sulfate–polyacrylamide gel electrophoresis on a 10% acrylamide gel and then electrotransferred onto a polyvinylidene difluoride membrane. Membranes were incubated with primary antibodies, including rabbit monoclonal phospho-IKKα/β antibody (1:1000), rabbit monoclonal IKK β antibody (1:1000), rabbit monoclonal p65 antibody (1:1000), rabbit monoclonal phospho-NF-κB p65 antibody (1:1000), rabbit monoclonal phospho-IκBα antibody (1:1000), and rabbit monoclonal IκBα antibody (1:1000). Immunoreactive bands were visualized by chemiluminescent HRP Antibody Detection Reagent and imaged using Image Lab software.

### ELISA

The levels of IL-17A, β-endorphin, serotonin, dopamine, dynophin, encephalin, noradrenaline, and IFN-γ in blood were determined using the commercially available ELISA kits. Optical density at a wavelength of 450 nm was determined with a microtiter plate reader.

### Immunohistochemistry

Spleens and lymph nodes were fixed in 4% paraformaldehyde for 12 h, embedded in paraffin, and cut into 4-μm sections. Then, these sections were deparaffinized and rehydrated with a series of gradient concentrations of alcohol, and 3% hydrogen peroxide was used to block the activity of endogenous peroxidase. Antigen retrieval was achieved by heating the tissue block in 0.01 mol/L sodium citrate buffer and washed with PBS. Non-specific binding was blocked with 5% bovine serum albumin. Each section was then incubated with certain concentrations of antibodies recommended by the manufacturers: anti-rat β-endorphin (the β-endorphin antibody powder was dissolved into a 500 μg/mL solution for storage and was then diluted into 10 μg/mL before use) (1:50), anti-rat CD4 (1:50), anti-rat CD8 (1:50), anti-rat granulocyte (HIS48) (1:20), and anti-rat IL-17A (1:50) antibodies, followed by incubation with the horseradish peroxidase-labeled secondary antibody. Finally, the sections were subjected to staining with a diaminobenzidine substrate-chromogen solution, followed by staining with hematoxylin.

### Cell Sorting and Immunofluorescence Staining

Splenic suspensions were sorted on an EasySep^TM^ magnet using the MojoSort Rat Isolation Kit to select T lymphocytes. The purity of cells enriched from spleen populations was consistently higher than 90%, evidenced by flow cytometry. Then the purified CD4^+^ T cells were washed with PBS, fixed in 4% paraformaldehyde for 30 min, and washed again with PBS. Cells were treated with 1% Triton X-100 for 10 min and washed twice. Cells were incubated with β-endorphin (1:50) antibody, anti-CD3 (1:50), anti-granulocyte (HIS48) (1:20), or anti-IL-17A (1:50). Next, cells were incubated with the fluorescence-conjugated secondary antibody in the dark. Finally, the cells were stained with DAPI and examined under the fluorescent microscope. In the other experiment, CD4^+^ T cells sorted from spleen were stimulated with plate-bound CD3, CD28 antibodies, and Brefeldin A solution (10 μg/mL), followed by treatment with IL-17A (50 ng/mL, 12 h). The expression of β-endorphin in CD4^+^ T cells was examined as above described.

### Statistical Analysis

All results were analyzed using GraphPad Prism version 7.0 and expressed as mean ± SD. The Student *t*-test was used to analyze the normally distributed independent data. Multiple measurements of normally distributed data were assessed by the one-way analysis of variance. *P* < 0.05 was considered statistically significant.

## Results

### EA Stimulation Relieves the Pain of BPRA in Rats by Up-Regulating β-Endorphin

**FIGURE 1:**
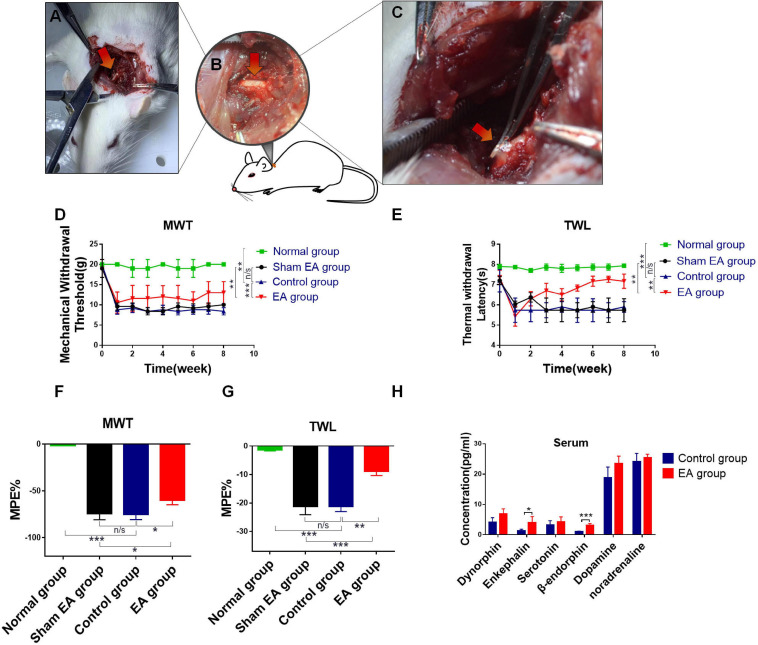
Evaluation of analgesic effect after EA treatment in BPRA model. **(A–C)** Establishment of rat brachial plexus root avulsion (BPRA) model. **(D–G)** Rats were divided into four groups, including normal group (healthy rats), control group (BPRA rats), sham EA group (needles were inserted into sham acupoints—0.8 cm beside left C5–C7 spinous, without electrical output), and EA group (BPRA rats received electroacupuncture therapy). The EA/sham EA treatment was carried out once a day for 2 months. Electron von Frey test and Hargreaves test were applied on rats in four groups during the EA treatment. The maximal possible effect (MPE) was calculated. A decrease in thermal withdrawal latencies was observed in all groups, whereas the EA group gradually recovered. **(H)** Concentration of analgesic-related protein in serum from control and EA groups was detected using ELISA kit, *n* = 5. Data are shown as mean ± SD, *p < 0.05, **p < 0.01, ***p < 0.001.

Animal models are the basis of scientific research. Therefore, we first generated the BPRA rat model in our study (Figures 1A–C). Rats were divided into four groups, including normal group (healthy rats), control group (BPRA rats), sham EA group (needles were inserted into sham acupoints—0.8 cm beside the left C5–C7 spinous, without electrical output), and EA group (BPRA rats received EA therapy) as described in our previous study (Hou et al., 2020). Surgical rats (control and EA group) showed signs of inactivity, such as slow movement and numbness. We assessed the nociceptive behavior using von Frey filaments and the Hargreaves test, which were applied to determine the paw withdrawal threshold and paw withdrawal latency, respectively. All of these two experiments are common indicators of pain tests. Rats in the control and EA groups showed a pretty low paw withdrawal threshold and long paw withdrawal latency, whereas rats in the normal group showed no abnormal activities in these indicators (Figures 1D–G). More and more studies have indicated that EA has a good analgesic effect on many types of pain (Spezia Adachi et al., 2018; Yen et al., 2019). The rats in the EA group received EA therapy (acupoints: 0.5 cm beside right C5–C7 spinous) once a day for 2 months (Supplementary Figure S1). We found that EA therapy could remarkably increase the paw withdrawal threshold and prolong the paw withdrawal latency compared with the control group (Figures 1D–G), which was consistent with the good analgesic effect of EA in previous research. By contrast, rats in the sham EA group that received sham EA therapy (sham acupoints: 0.8 cm beside the left C5–C7 spinous, without electrical output) had no such effect. Several studies have shown that analgesia can be achieved by the production of secreted neurotransmitters, such as dynorphin, enkephalin, serotonin, β-endorphin, dopamine, and noradrenaline. Among these, β-endorphin is an endogenous compound, and it can bind to morphine receptors, resulting in analgesic and euphoric effects as morphine and opiates (Huang et al., 2017; Luan et al., 2017). In this study, we assessed the relationship between EA and the aforementioned pain-related neurotransmitters by ELISA. We found that EA could dramatically increase the level of β-endorphin (Figure 1H).

### The Immune System of BPRA Rats Is Regulated by EA Stimulation

**FIGURE 2:**
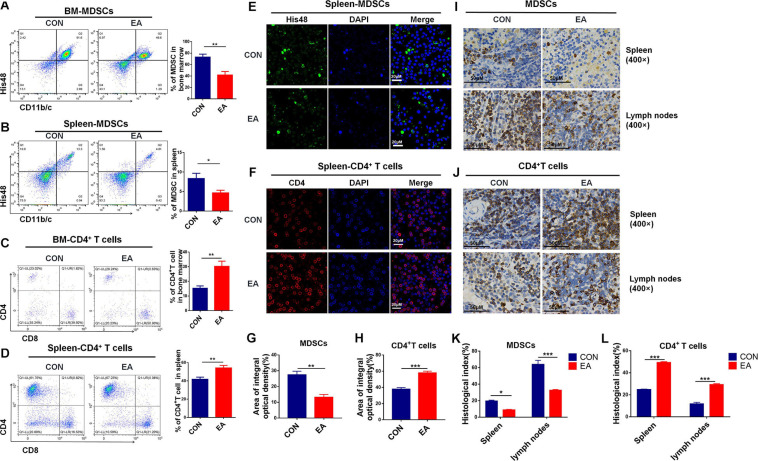
Effect of EA treatments on collective immunity of mice. **(A–D)** The population of MDSCs and T cells was detected with flow cytometry in rats’ spleen and bone marrow from the control and EA groups, respectively, *n* = 7. Data are shown as mean ± SD, *p < 0.05, **p < 0.01. **(E–H)** IF was used to detect MDSCs (His48) and CD4^+^ T cells in rat spleen. **(I–L)** Immunohistochemistry was used to detect MDSCs (His48) and CD4^+^ T cells in the spleen of rats, and the results were observed under a microscope (×400), *n* = 3. Data are shown as mean ± SD, *p < 0.05, **p < 0.01, ***p < 0.001.

Accumulating evidence shows that EA exhibits a good regulatory effect on the immune system under various diseased conditions, such as asthma, rheumatoid arthritis, and so on (Li et al., 2017; Wu et al., 2018). In our present study, the pain was significantly reduced in BPRA rats treated with EA, and we further clarified how the immune system was changed upon EA stimulation. Pain is often closely related to inflammation. Neuropathic pain can be caused by chronic inflammation. CD4^+^ T cells, CD8^+^ T cells, natural killer (NK) cells, macrophages, and myeloid-derived suppressor cells (MDSCs) usually are involved in and play an important role in the inflammatory microenvironment (Taylor and Colgan, 2017). Therefore, we detected these aforementioned cells to assess the relationship between EA and the immune system in this study. Flow cytometry showed that the number of MDSCs could be remarkably decreased by EA treatment in the spleen and bone marrow. Meanwhile, the number of CD4^+^ T cells, not CD8^+^ T cells, could be markedly increased in bone marrow and especially in splenocytes (Figure 2 and Supplementary Figures S2A–J). Besides, NK cells, dendritic cells (DCs), and macrophages were increased, while such changes had no significance (Supplementary Figures S2A–F). Moreover, immunofluorescence (IF) and immunohistochemistry (IHC) were also applied to examine the distribution of MDSCs and T cells. Consistent with the flow results, the number of MDSCs was significantly reduced, whereas CD4^+^ T cells were remarkably increased in spleen and lymph nodes (Figures 2E–L). Moreover, the level of IFN-γ, as the main activation marker cytokine of T helper cells, was also increased in serum (Supplementary Figure S2K). It seemed that CD4^+^ T cells would play an important role in the BPRA rat model due to EA stimulation. Therefore, we hypothesized that the analgesic effects of EA were associated with the increased CD4^+^ T cell infiltration and the enhanced level of β-endorphin.

### β-Endorphin Is Released by CD4^+^ T Lymphocytes After EA Treatment

**FIGURE 3:**
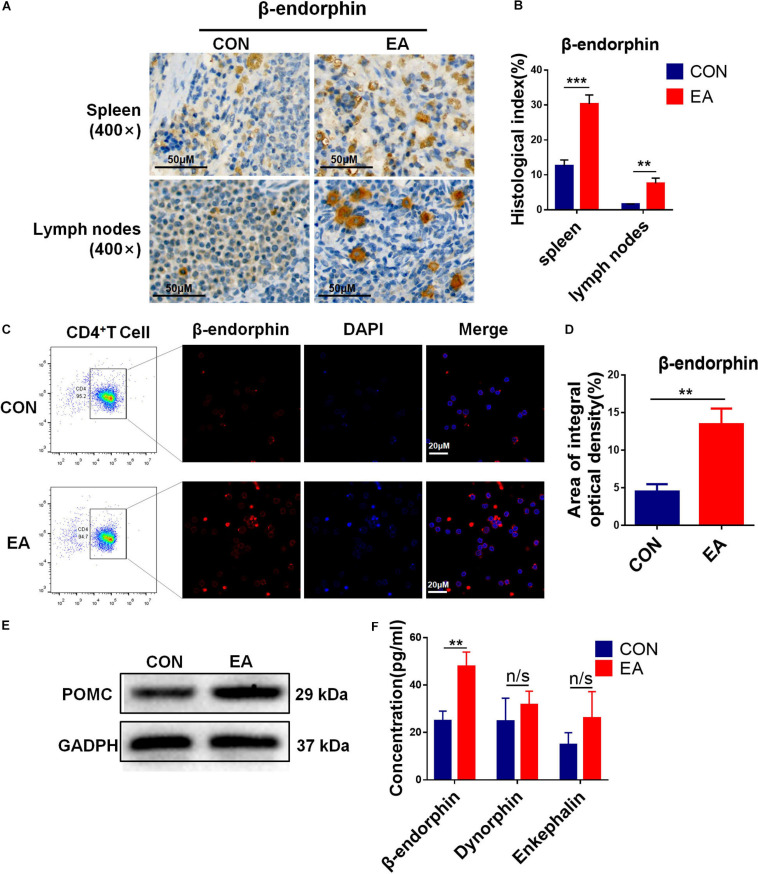
EA stimulation promotes the secretion of β-endorphin by CD4^+^ T cells. **(A,B)** IHC was used to detect the expression of β-endorphin in the spleen and lymph nodes of rats, and the results were observed under a microscope (×400), *n* = 3. Data are shown as mean ± SD, ^∗∗^*p* < 0.01, ^∗∗∗^*p* < 0.001. **(C–E)** CD4^+^ T cells in rat spleen were sorted by a magnet with the MojoSort Rat Isolation Kit, and their purity was confirmed to be greater than 90% by flow cytometry. CD4^+^ T cells sorted from spleen were stimulated with CD3, CD28 antibodies, and Brefeldin A solution. IF was used to detect the expression of β-endorphin in CD4^+^ T cells sorted from rat spleen, and Western blotting analysis was performed to detect the protein expression of POMC in them, *n* = 3. Data are shown as mean ± SD, ^∗∗^*p* < 0.01. **(F)** The concentration of β-endorphin, dynorphin, and enkephalin in the sorted CD4^+^ T cells stimulated with CD3, CD28 antibodies from the control and EA groups were detected using ELISA kit, respectively, *n* = 6. Data are shown as mean ± SD, ^∗∗^*p* < 0.01.

**FIGURE 4:**
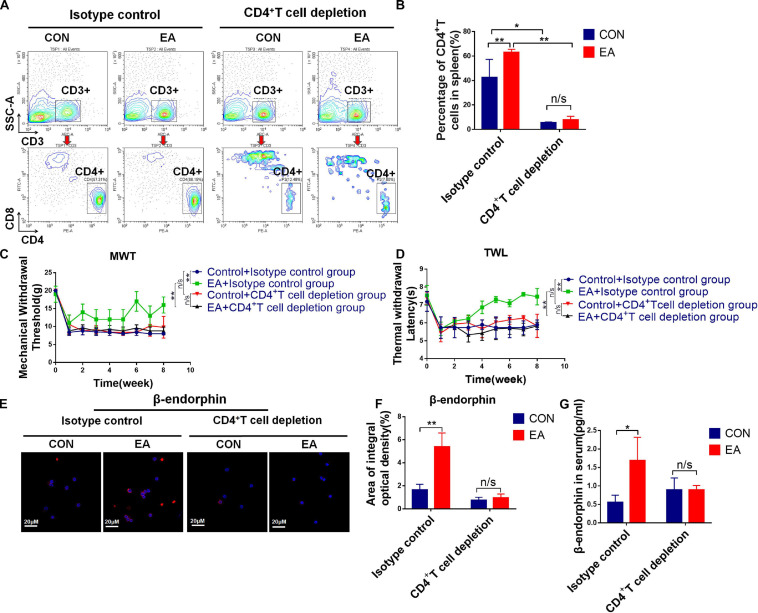
The secretion of β-endorphin is mediated by CD4^+^ T cells under EA therapy. **(A,B)** Rats were treated with 5 mg/kg anti-CD4 antibody or control IgG by intraperitoneal injections twice a week for 2 months to deplete CD4^+^ T cells followed by EA treatment. Knockout efficiency was then measured by flow cytometry using CD3 and CD4 antibody, *n* = 3. Data are shown as mean ± SD, ^∗^*p* < 0.05, ^∗∗^*p* < 0.01. **(C,D)** Electron von Frey test and Hargreaves test were applied to rats after acupuncture treatment with CD4^+^ T cell depletion. The data were analyzed in week 8, *n* = 3. Data are shown as mean ± SD, ^∗∗^*p* < 0.01. **(E,F)** IF was used to detect the expression of POMC/β-endorphin in the spleen after the depletion of CD4^+^ T cells, *n* = 3. Data are shown as mean ± SD, ^∗∗^*p* < 0.01. **(G)** After CD4^+^ T cell depletion, the concentration of β-endorphin in serum was detected using ELISA kit, *n* = 3. Data are shown as mean ± SD, ^∗^*p* < 0.05.

Based on our results, EA exerted a good modulatory effect on the immune system, especially on CD4^+^ T cells. To further confirm whether CD4^+^ T cells (not CD8^+^ T cells) had an analgesic effect on the BPRA rats, we intraperitoneally injected CD8 monoclonal antibodies intraperitoneally into these rats and exhausted CD8^+^ T cells. It was found that the presence or absence of CD8^+^ T cells had little effect on the analgesic effect of EA treatment (Supplementary Figure S3). Therefore, we continued to localize effector cells of the EA analgesic effect on CD4^+^ T cells. Studies have reported that EA can stimulate the release of β-endorphin by immune cells. We found that the expression of β-endorphin was increased in both spleen and lymph nodes when the model rats received EA treatment (Figures 3A,B). Subsequently, we determined whether CD4^+^ T cells played a predominant role. As we demonstrated that EA therapy could stimulate the infiltration of more CD4^+^ T cells (Figure 2), we next explored the relationship between CD4^+^ T cells and β-endorphin. The spleen of the model rat was dissected, the CD4^+^ T cells were separated by magnetic bead, and flow cytometry showed that the purity of the CD4^+^ T cells was more than 90%. We used the IF assay to detect the expression of β-endorphin (Supplementary Figure S4A and Figure 3C). We found that the level of β-endorphin in CD4^+^ T cells of BPRA rats was significantly increased after EA treatment (Figures 3C,D). Moreover, Western blotting analysis and ELISA exhibited that the expression of POMC [pro-opiomelanocortin (POMC) is the precursor of β-endorphin, which is the “raw material” for the synthesis of β-endorphin] at the protein level was significantly increased in CD4^+^ T cells after EA therapy (Figures 3E,F). Besides, more pain-related factors were detected in peripheral immune organ (spleen), such as serotonin-receptor (HTR1A, HTR2A), prodynorphin (PDYN), dynorphin receptors (OPRK1), POMC, μ-opioid receptor (MOR), dopamine receptor (DRD1, DRD2), and adrenaline receptor (α2-AR). The expressions of these factors at the mRNA level were examined by RT-qPCR, and we found that the expressions of POMC, PDYN, and MOR were increased after EA therapy. However, the up-regulation of POMC was most obvious (Supplementary Figure S4B). This result was consistent with the expression of POMC at the protein level in purified CD4^+^ T cells and also in agreement with several previous reports that immune cells can secret opioid peptides under inflammatory conditions. In the absence of such cells, no secretion of opioid peptides has been observed (Plein and Rittner, 2018). To further verify such findings, we depleted the CD4^+^ T cells in BPRA rats also using monoclonal antibodies (Figures 4A,B and Supplementary Figures S5A–C) and first observed whether the analgesic effect of EA still existed. Consistently, when the CD4^+^ T cells were exhausted, the analgesic effect of EA on the model rat was almost completely abolished (Figures 4C,D), suggesting that EA-mediated analgesia in BPRA rats was indeed dependent on these CD4^+^ T cells. Next, we further examined whether the release of β-endorphin also depended on T cells. The data of IF, ELISA, and IHC indicated that the release of β-endorphin was significantly reduced when the CD4^+^ T cells were depleted (Figures 4E–G and Supplementary Figure S5D). This finding suggested that the analgesic effects of EA in the model rats were mediated by β-endorphin secreted by CD4^+^ T cells.

### IL-17 Contributes to the Release of β-Endorphin in CD4^+^ T Lymphocytes

**FIGURE 5:**
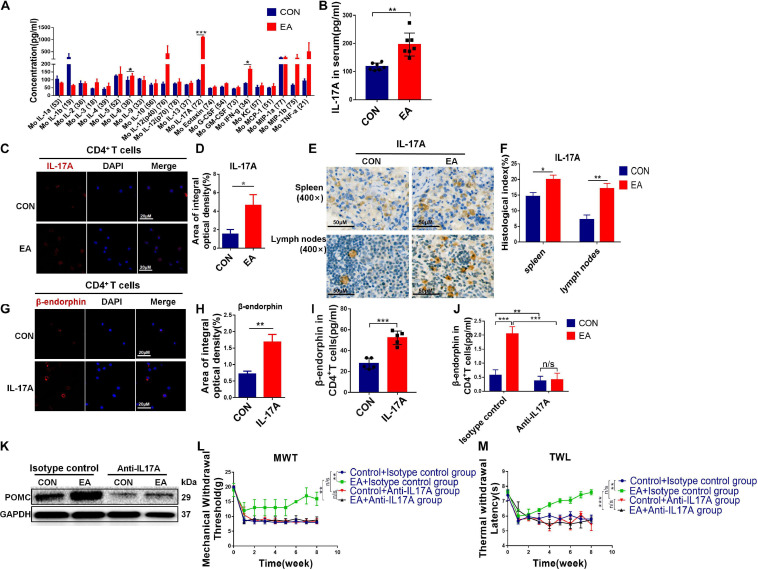
IL-17 promotes secretion of β-endorphin from CD4^+^ T cells. **(A)** Cytokine array was applied to analyze the quantification of inflammatory cytokines in serum from the control and EA groups, *n* = 7. Data are shown as mean ± SD, ^∗∗^*p* < 0.01. **(B)** ELISA was applied to analyze the quantification of IL-17A in serum from both control and EA groups, *n* = 7. Data are shown as mean ± SD, ^∗∗^*p* < 0.01. **(C,D)** CD4^+^ T cells sorted from spleen were stimulated with CD3, CD28 antibodies, and Brefeldin A solution. IF was used to detect the expression of IL-17A in CD4^+^ T cells sorted from rat spleen, *n* = 3. Data are shown as mean ± SD, ^∗^*p* < 0.05. **(E,F)** IHC was used to detect the expression of IL-17A in the spleen and lymph nodes of rats, and the results were observed under a microscope (×400), *n* = 3. Data are shown as mean ± SD, ^∗^*p* < 0.05, ^∗∗^*p* < 0.01. **(G,H)** Activated CD4^+^ T cells were treated with IL-17A for 12 h. IF was used to detect β-endorphin expression in CD4^+^ T cells sorted from rat spleen, *n* = 3. Data are shown as mean ± SD, ^∗∗^*p* < 0.01. **(I)** After IL-17A was added in stimulated CD4^+^ T cells, the concentration of β-endorphin in CD4^+^ T cells was detected using ELISA kit, *n* = 5. Data are shown as mean ± SD, ^∗∗∗^*p* < 0.001. **(J)** Rats were treated with 10 mg/kg anti-IL-17A antibody, or control mouse IgG by intraperitoneal injections once a week for 2 months to neutralize IL-17A before EA treatment. CD4^+^ T cells were sorted from the spleen, and the concentration of β-endorphin in CD4^+^ T cells was detected using an ELISA kit, *n* = 3. Data are shown as mean ± SD, ^∗∗^*p* < 0.01, ^∗∗∗^*p* < 0.001. **(K)** After IL-17A neutralization, CD4^+^ T cells were sorted from the spleen, and Western blotting analysis was applied to detect the protein expression of POMC in CD4^+^ T cells in both control and EA groups. **(L,M)** Electron von Frey test and Hargreaves test were applied to rats after EA treatment with IL-17A depletion. The data were analyzed in week 8, *n* = 3. Data are shown as mean ± SD, ^∗∗^*p* < 0.01, ^∗∗∗^*p* < 0.001.

As mentioned earlier, pain is closely related to inflammation. Therefore, we examined a range of cytokines associated with inflammation via cytokine array. IL-17 cytokines play a critical role in immunity and inflammatory diseases. IL-17A is the founding member of the IL-17 family, which promotes inflammation and host defense (Li et al., 2019). We found that IL-17A was significantly up-regulated after EA treatment (Figure 5A). ELISA and IHC exhibited similar results (Figures 5B,E,F). Subsets of helper T cells (CD4^+^ T cells), Th17, secrete IL-17, which is also the major source of IL-17. Besides, IL-17 can also in turn bind to surface receptors of CD4^+^ T cells (Luo et al., 2014). This might explain why the titer of IL-17A was significantly down-regulated when we depleted CD4^+^ T cells (Supplementary Figures S6A–C). Moreover, we purified CD4^+^ T cells, and IF assay showed that the content of IL-17A in the EA group was remarkably higher compared with the control group (Figures 5C,D). According to our results, EA treatment could increase the infiltration of CD4^+^ T cells and promote them to release more β-endorphin, leading to an increased titer of IL-17A. Therefore, we attempted to confirm the relationship among these three factors. The purified CD4^+^ T cells were incubated in the presence of IL-17A for 12 h, and then the content of β-endorphin was determined by IF and ELISA (Figures 5G–I). We found that the release of β-endorphin was significantly increased when cells were cultivated in the presence of IL-17A, indicating that the release of β-endorphin by CD4^+^ T cells was mediated by IL-17A. However, on the other hand, if monoclonal antibodies were used to deplete IL-17A in BPRA rats (Supplementary Figures S6D–F), the up-regulation of β-endorphin and POMC in CD4^+^ T cells mediated by EA was almost completely abolished, and its analgesic effect was nearly lost as well (Figures 5J–M), further explaining that the presence of IL-17A was the basis for EA to exert analgesia. Taken together, the absence of any link of IL-17A-CD4^+^ T cell–β-endorphin axis could lead to the destruction of EA-mediated analgesia.

### IL-17A Induces the Release of β-Endorphin by Activating the NF-κB Signaling Pathway

**FIGURE 6:**
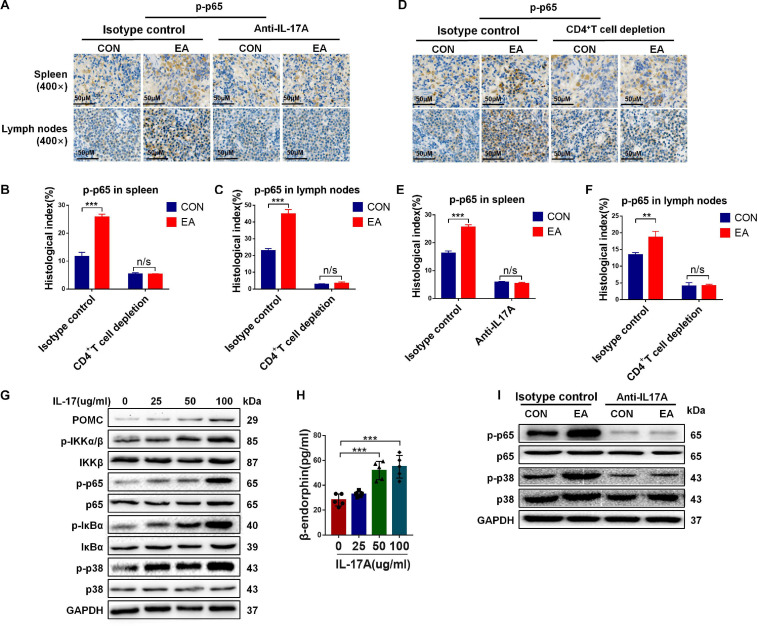
The activation of NF-κB signaling pathway induced by IL-17 promotes β-endorphin secretion from CD4^+^ T cells. **(A–F)** IF was used to detect the protein expression of phosphorylated p65 in the spleen and lymph nodes of rats before and after the depletion of CD4^+^ T cells or treatment with IL-17A, *n* = 3. Data are shown as mean ± SD, ^∗∗^*p* < 0.01, ^∗∗∗^*p* < 0.001. **(G)** CD4^+^ T cells sorted from spleen were stimulated with CD3, CD28 antibodies, and Brefeldin A solution, followed by treatment with IL-17A of different concentrations (0, 25, 50, and 100 μg/mL) for 12 h. Western blotting analysis was applied to detect the protein expression of POMC, p-IKKα/β, IKKβ, p65, p-p65,IκBα, p-IκBα, p38, and p-p38. **(H)** After IL-17A treatment with stimulated CD4^+^ T cells (0, 25, 50, and 100 μg/mL), the concentration of β-endorphin in serum was detected using ELISA kit, *n* = 5. Data are shown as mean ± SD, ^∗∗∗^*p* < 0.001. **(I)** After IL-17A was depleted, the Western blotting analysis was applied to detect the protein expressions of p65, p-p65, p38, and p-p38.

**FIGURE 7:**
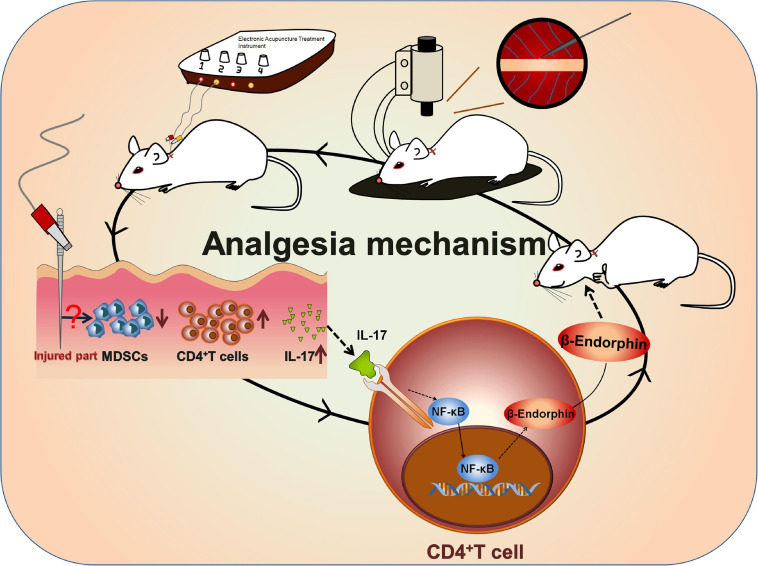
Mechanism of EA-induced analgesia. A rat BPRA model was established, and EA treatment was applied to the model rat 1 week after surgery for 2 months. The infiltration of CD4^+^ T cells was increased after EA stimulation, which subsequently promoted the release of IL-17 probably by inhibiting MDSCs. Then, IL-17 in turn promoted the secretion of β-endorphin via activating the NF-κB signaling pathway in CD4^+^ T cells. In summary, IL-17–CD4^+^ T cell–β-endorphin axis played a vital role in EA-induced analgesia of BPRA rats.

A previous study has reported that NF-κB can mediate IL-17 downstream signaling in various types of mammalian cells (Mohammed et al., 2018). In the present study, we hypothesized that IL-17A promoted the secretion of β-endorphin by activating the NF-κB signaling pathway. To test our hypothesis, the level of p65, as one member of the NF-κB family, and its phosphorylation level p-p65 were determined by IHC in the spleen and lymph nodes of BPRA rats. The results showed that the expression of p-p65 was remarkably enhanced after EA stimulation, and when IL-17A was depleted, the up-regulation was almost completely abolished (Figures 6A–C). The resembling results were also found in CD4^+^ T cells-depleted rats (Figures 6D–F). To further test whether the NF-κB signaling pathway was involved in IL-17-mediated effects, the CD4^+^ T cells were collected from splenocytes after treatment with IL-17A at different concentrations (25, 50, and 100 μg/mL) for 12 h. Activation of the NF-κB signaling pathway involves the phosphorylation and up-regulation of p-IKKα/β, p-IκBα, p-p65, and p-p38 (Hwang et al., 2018). Therefore, in our study, we also detected these phosphorylated markers of the NF-κB signaling pathway. It was found that the levels of p-IKKα/β, p-IκBα, p-p65, and p-p38 were significantly increased after IL-17A addition, indicating that the NF-κB signaling pathway was activated by IL-17A (Figure 6G), which was consistent with the previous data. Moreover, with the increase of IL-17A concentration, the phosphorylated levels of IKKα/β, p65, IκBα, and p38 were also up-regulated, showing that the NF-κB signaling pathway was activated in an IL-17A concentration-dependent manner (Figure 6G). Moreover, the protein expression of POMC was elevated by the increasing IL-17A as well (Figure 6G), which was consistent with the enhanced β-endorphin detected by ELISA assay (Figure 6H). However, when IL-17A was neutralized in BPRA rats, the up-regulation of phosphorylated p65 and p38 was almost completely reversed, evidenced by Western blotting analysis (Figure 6I). Collectively, all the above results demonstrated that IL-17A induced the release of β-endorphin by activating the NF-κB signaling pathway in CD4^+^ T cells of the BPRA rat model (Figure 7).

## Discussion

Brachial plexus root avulsion-caused pain is largely featured by the quick onset of pain and the periodic sharp paroxysms of neuropathic pain. In this study, a BPRA rat model was established to assess the effect of EA treatment on BPRA rats. Two behavior assessments, von Frey filaments and the Hargreaves test, were applied on the contralateral side of BPRA rats. The contralateral side rather than ipsilateral (injured) side was chosen mainly for two reasons. First, the BPRA-caused pain may be distant to the site of the lesion either on the ipsilateral or contralateral side due to the injury-caused magnified peripheral sensitization of nociceptors (Rubens et al., 2003). In other words, although the contralateral limb was non-injured, the pain threshold of the contralateral side would be reduced, whereas the sensitivity to pain was enhanced by the BPRA-caused neuropathic pain on the ipsilateral side. Second, clinical patients with BPRA suffered from periodic sharp paroxysms of pain, causing them to cry out and even grab their injured arms (Parry, 1980). Similar situation happened on our BPRA rats. Approximately 50% of the BPRA rats showed varying degrees of autonomic behavior (biting the digits of injured limbs); even in some of them, all the digits of injured limb were involved (Li et al., 2018; Shen et al., 2019). Therefore, there was no internal control within the study because of the objective limitation. For this reason, the pain threshold on the rats under sham surgery was assessed as control in our study.

Electroacupuncture, a traditional complementary and alternative therapy, is one of the most popular analgesic methods. Many studies have reported pain relief with EA using rat models, while only very few rat models of BPRA have been established (Baddack-Werncke et al., 2017; Ben-Shaanan et al., 2018). In the present study, we hypothesized that there was a close interplay between the analgesic effect of EA and immune regulation in the rat model of BPRA. EA excites cutaneous and muscle afferent nerve fibers, which is believed to activate the endogenous pain-controlling system and the release of β-endorphin (Basso et al., 2016). In our study, we found that the pain was significantly reduced in the model rat upon EA treatment (Figure 1). Increasing evidence has shown that EA can regulate the immune system of the inner body (Hughes et al., 2013). T cells, MDSCs, macrophages, DCs, and NK cells were detected in BPRA rats. We observed that the number of MDSCs was significantly decreased, whereas that of T cells, especially CD4^+^ T cells (not CD8^+^ T cells), was dramatically elevated (Figure 2A and Supplementary Figure S2), and when the CD4^+^ T cells were depleted, the population of MDSCs was markedly enhanced (Supplementary Figure S5D). In the tumor microenvironment, MDSCs usually exert immunosuppressive effects by inhibiting the immune activity of T cells to promote tumor growth. Recent research shows that MDSCs can modulate the functions of the reward system, especially the ventral tegmental area, leading to the activation of tumor growth. Such activation is regulated via the sympathetic nervous system, which is featured by a weakened noradrenergic input to a major immunological site, bone marrow. MDSCs become less immunosuppressive following activation of the reward system, suggesting that MDSCs are closely related to the neuroendocrine system (Hammer et al., 2016). Therefore, it was also possible that the significant reduction of MDSCs in our study might be directly involved in the regulation of neuroimmunity other than indirect effects on T cells. However, such a hypothesis needs to be further validated in our future work.

Under inflammatory conditions, pain is mediated by endogenous opioids derived from immune cells. Inflammation-triggered somatic pain is first weakened by opioid-producing inflammatory cells, including neutrophils and macrophages, and then it is abolished by opioid-producing T lymphocytes recruited to the inflammation site a few days later (Zheng et al., 2014). In the present study, we found that the infiltration of CD4^+^ T cells was significantly enhanced. Although the number of macrophages was also increased, the changes were not obvious after the model rats received EA treatment for 2 months, which was consistent with the aforementioned findings (Supplementary Figure S2). T cell-derived opioids play a key role in the control of inflammatory pain. However, β-endorphin has already been found in T lymphocytes by using antibody-based methods (Basso et al., 2016). Based on this finding, we detected a range of analgesic neurotransmitters, including opioids, dynorphin, enkephalin, β-endorphin, and other substances (Figure 1 and Supplementary Figure S4B). Among these neurotransmitters, the expressions of β-endorphin and its precursor POMC were significantly increased after EA stimulation, which was also consistent with previous conclusions. However, the nature of opioids derived from T cells remains a debating topic, no matter in mice or rats.

Next, when the CD4^+^ T cells were depleted, we found that the expression of β-endorphin was significantly decreased. Moreover, the analgesic effect was reversed in the model rats (Figures 4C,D). The aforementioned results indicated that β-endorphin-mediated endogenous regulation of pain by CD4^+^ T lymphocytes would be entirely abolished when CD4^+^ T lymphocytes were depleted (Figure 4 and Supplementary Figure S5). These data suggested that the opioid peptides were released in chronically inflamed tissue, extending our knowledge gained from acute inflammatory models. However, it remained unclear how β-endorphin was increased by CD4^+^ T lymphocytes. Growing evidence has shown that IL-17 plays a critical role in the recruitment of inflammatory cells to injury sites, leading to the production of a lineage of helper T cells (CD4^+^ T lymphocytes), Th17 cells. A previous study also exhibits that IL-17 is monophasically expressed in degenerating nerves after chronic constriction injury, and IL-17-positive T cells are detectable within the endoneurium of injured nerves caused by inflammation (Amatya et al., 2017). As a proinflammatory factor, the role of IL-17 in analgesia remains controversial. Some studies have indicated that in the absence of IL-17 (IL-17^–/–^), the inflammatory response to the injured sites (partial sciatic nerve ligation) is significantly weakened, and the behavioral hypersensitivity to pain is suppressed. The mechanism underlying such decreased behavioral hypersensitivity could be attributed to the attenuated infiltration of inflammatory cells, as well as the altered cytokine profiles observed at the site of nerve injury in IL-17-deficient mice. Moreover, IL-17 deficiency has also been linked to reduced expressions of opioid peptides at the injured site (Day et al., 2014). However, the up-regulated IL-17 contributed to the infiltration of immune cells (CD4^+^ T lymphocytes) in the peripheral immune system (such as spleen and lymph node) in our research, and the increased expression of β-endorphin derived from CD4^+^ T cells could relieve the pain hypersensitivity. It should also be noted, however, that there are conflicting findings on the roles of IL-17 in inflammation-induced nerve dysfunction. These differences may be attributed to exposure to different immune microenvironments, and in this BPRA rat model, EA therapy modulated the immune system and promoted the system to “take advantage and avoid harm.” However, more detailed investigations are still necessary to clarify whether IL-17 is involved in the induction of the pain associated with peripheral neuropathy. For instance, depletion of IL-17A (IL-17A^–/–^) in the BPRA model rat could be carried out in our subsequent studies, which would be more reliable than using IL-17A monoclonal antibodies in this study. Up to date, our present data preliminarily implied that the IL-17A-CD4^+^ T cell–β-endorphin axis might exert a critical function in EA analgesia in the BPRA model.

However, after IL-17A binds to its receptor, numerous signaling intermediates are engaged to transduce downstream signaling, including NF-κB, MAPK, and CCAAT-enhancer-binding protein, to trigger the expressions of antibacterial peptides, proinflammatory chemokines, and cytokines, as well as matrix metalloproteinases (Jimi et al., 2019). As a member of the NF-κB family, p65 is a transcription factor. In our current study, we showed that NF-κB was activated in BPRA rats upon EA treatment. Moreover, the degree of NF-κB activation was closely related to the titer of IL-17A, indicating that when the concentration of IL-17A was increased, the protein phosphorylation in the NF-κB signaling pathway was also remarkably enhanced in purified CD4^+^ T cells. Meanwhile, with the increase of IL-17A concentration, the expression of β-endorphin and its precursors POMC also increased (Figure 6). However, when IL-17A of BPRA rats was depleted, not only the β-endorphin release of the rats was decreased sharply, but also the EA-mediated analgesia almost disappeared (Figures 5J–M). Besides, the activation of the NF-κB signaling pathway induced by IL-17A was also suppressed with its neutralization (Figure 6I). Therefore, based on our results, IL-17A triggered the NF-κB signaling pathway to induce the infiltration of CD4^+^ T cells, further promoting the release of β-endorphin (Figure 7).

## Conclusion

In summary, we, for the first time, found that the pain caused by BPRA surgery could be significantly relieved by EA treatment via modulating the immune system, which was dependent on the IL-17–CD4^+^ T cell–β-endorphin axis to achieve the analgesic effect. Besides, we also demonstrated that IL-17A increased β-endorphin secretion by activating the NF-κB signaling pathway in CD4^+^ T cells. Moreover, when CD4^+^ T cells or IL-17A was depleted, the release of β-endorphin was almost completely abolished, as well as the analgesic effect of EA. Collectively, our findings enriched the analgesic mechanism of peripheral opioids upon EA therapy in the BPRA rat model.

## Data Availability Statement

The original contributions presented in the study are included in the article/Supplementary Material. Further inquiries can be directed to the corresponding author/s.

## Ethics Statement

The animal study was reviewed and approved by the Ethics Committee of Shanghai University of Traditional Chinese Medicine.

## Author Contributions

ZX designed the study, performed animal work, performed and analyzed the flow cytometry experiments, immunofluorescence, and ELISA, wrote the manuscript, and funded the project. YZ carried out the animal behavioral experiments, cell isolations, RT-qPCR, and Western blot, analyzed the *in vitro* and *in vivo* data, and wrote the manuscript. JS generated the BPRA rat model and performed rat EA treatment. LS, XJ, and YH performed rat nociceptive (von Frey and Hargreaves) test and EA stimulation. CZ and XC conceived the project and analyzed and interpreted the data. YL and ZM carried out immunohistochemistry and generated the pictures of the stained sections. LW, CY, SZ, and CG guided the design and writing of the manuscript. JX designed the whole study, wrote and revised the manuscript, and funded the project. ML helped with the revision. All authors revised the manuscript drafts and read and approved the final manuscript.

## Conflict of Interest

The authors declare that the research was conducted in the absence of any commercial or financial relationships that could be construed as a potential conflict of interest.
