# Supplementary material for: Pain Relief Dependent on IL-17–CD4+ T Cell–β-Endorphin Axis in Rat Model of Brachial Plexus Root Avulsion After Electroacupuncture Therapy
Source: Front Neurosci. 2021 Feb 9;14:596780. doi: 10.3389/fnins.2020.596780 (PMC7901907; doi:10.3389/fnins.2020.596780)
Supplement: Supplementary file 1 [file Table_2.docx]

Supplementary Material

## Supplementary Figures


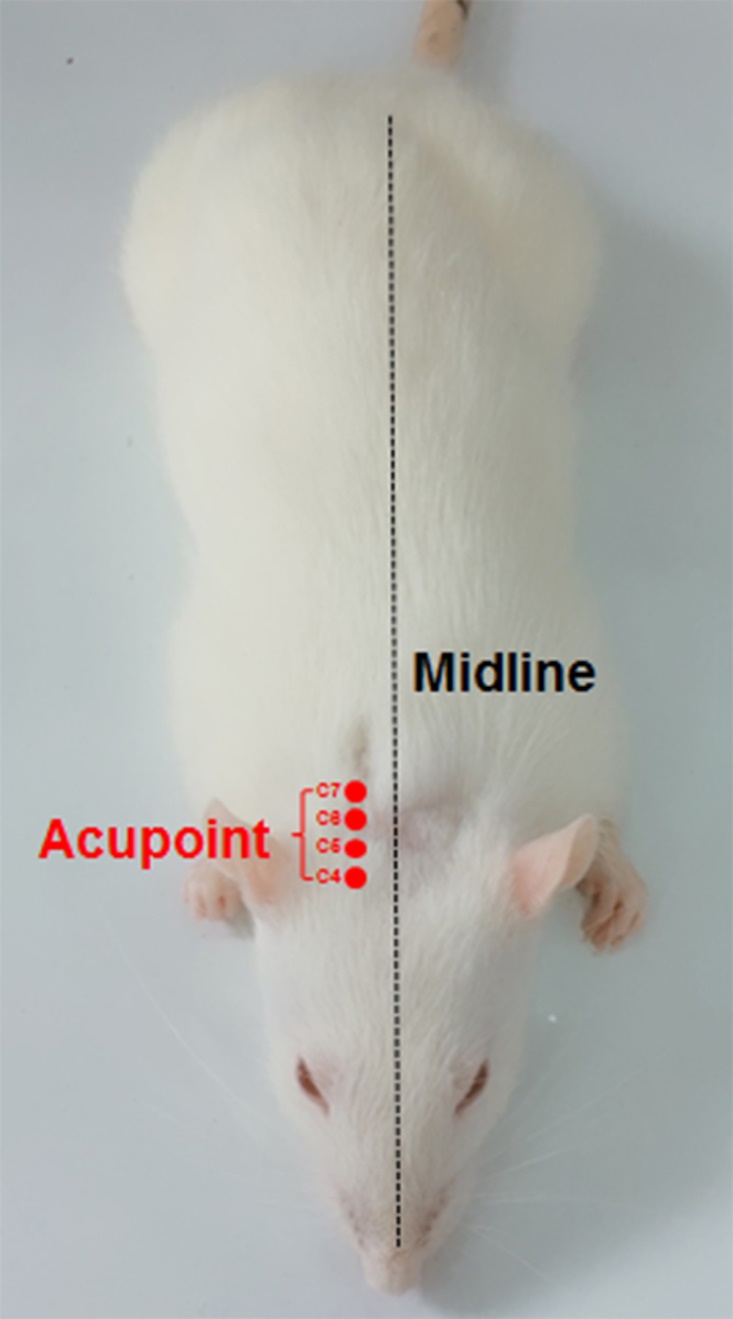


**Supplementary Figure S1**

The fur over the acupoint was removed and the exposed skin was cleaned before EA treatment. The acupoint was located in the vertex on the midline 0.5 cm beside C5-C7 spinous process.


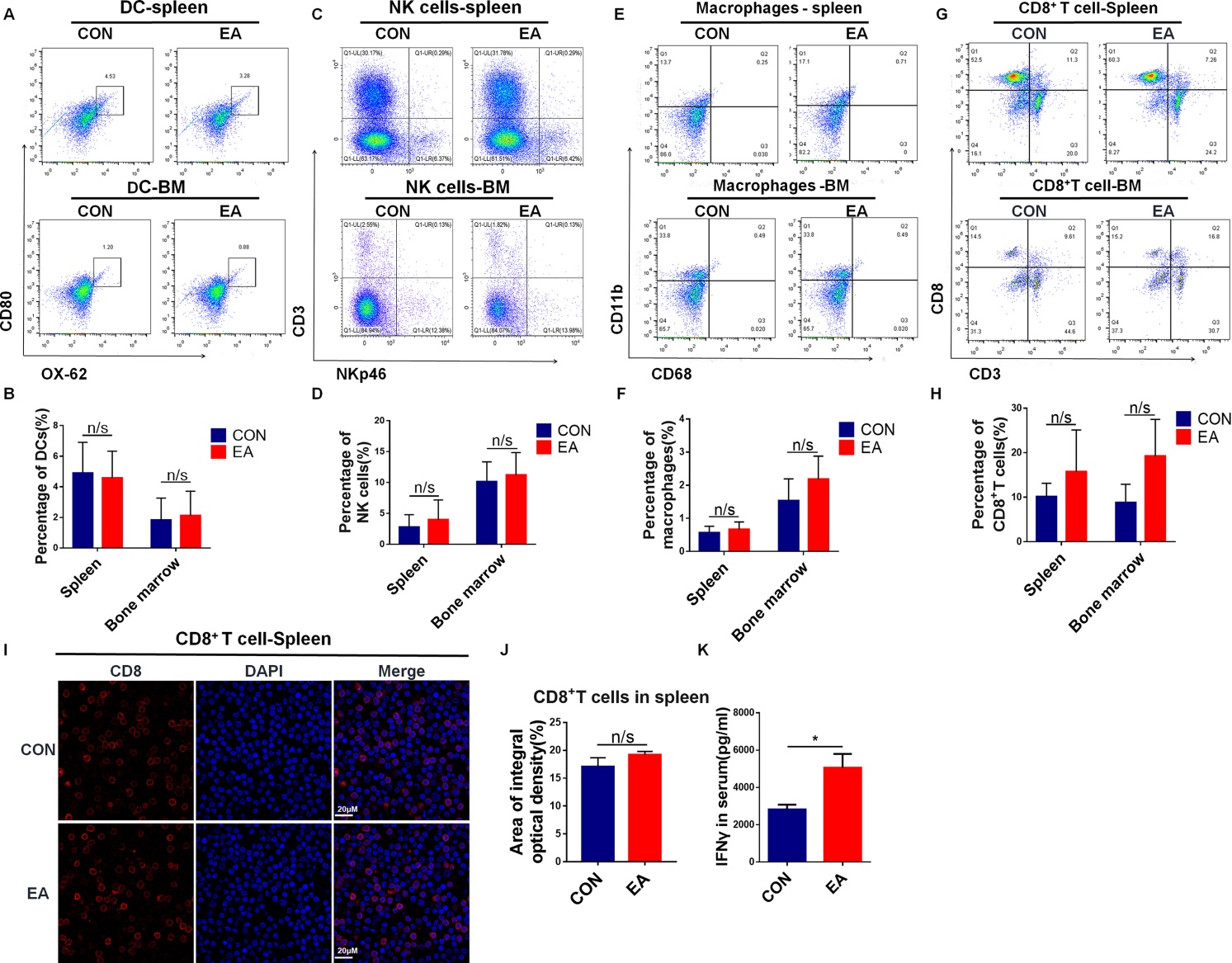


**Supplementary Figure S2**

**A-H** Flow cytometry for detection of DCs (CD80^+^ OX-62^+^), NK cells (CD3^-^ NKp46^+^), macrophages (CD11b^+^ CD68^+^) and CD8^+^ T cells in spleen and bone marrow of rats from control and EA group was applied, n=6. Data are shown as mean ± SD. **I-J** Immunofluorescence was used to detect CD8^+^ T cells in rat spleen, n=3. Data are shown as mean ± SD. **K** Concentration of IFN-γ in serum after EA treatment was detected using ELISA kit, Data are shown as mean ± SD, **p*<0.05.


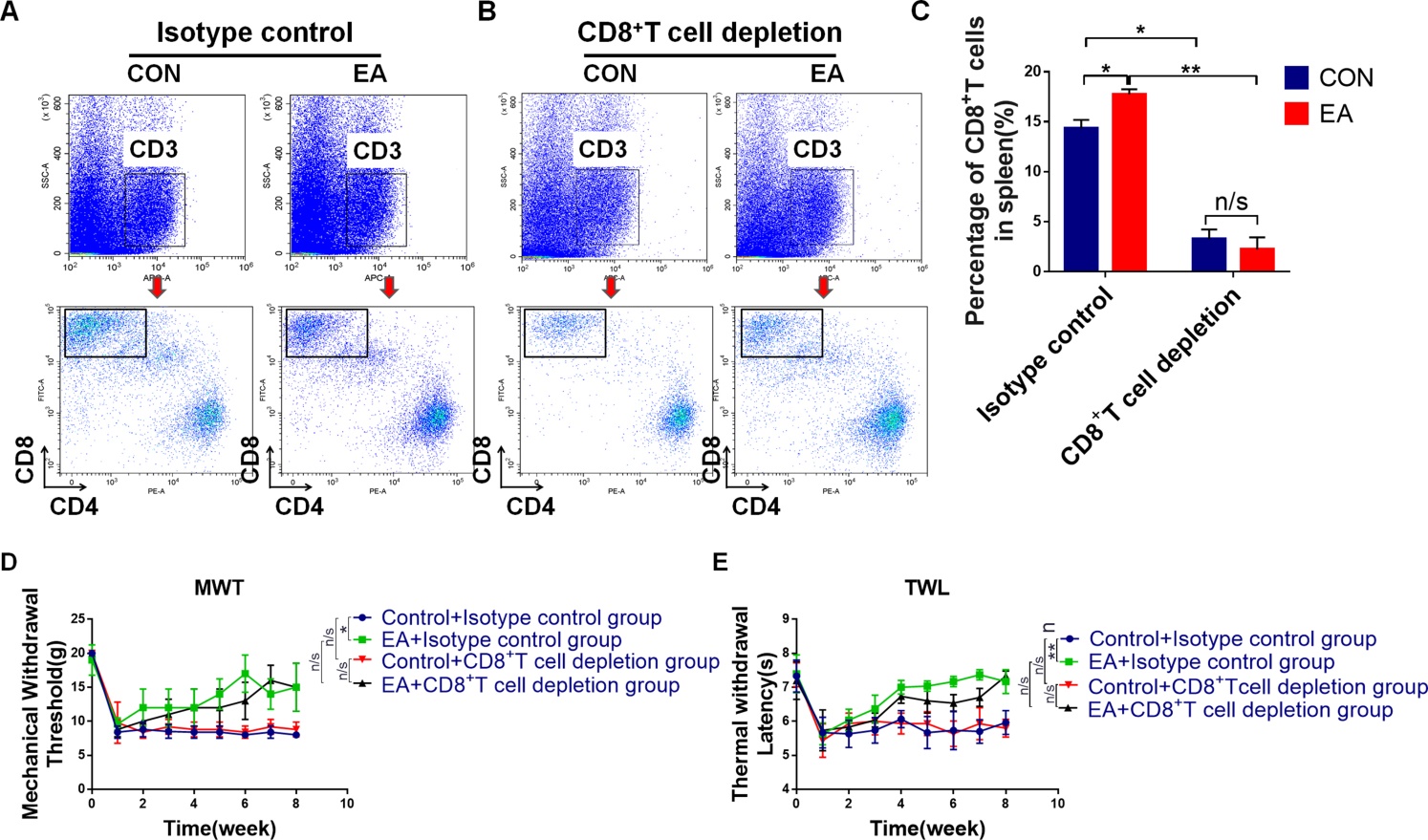


**Supplementary Figure S3**

**A-C** Anti-CD8^+^ T cell antibody was applied to deplete CD8^+^ T cells before EA treatment; and the effect in spleen was detected by flow cytometry, n=3.Data are shown as mean ± SD, **p*<0.05,***p*<0.01.**D-E** Electron von Frey test and Hargreaves test were applied on rats after acupuncture treatment with CD8^+^ T cells depletion. The data was analyzed on week 8, n=3. Data are shown as mean ± SD,**p*<0.05, ***p*<0.01**.**


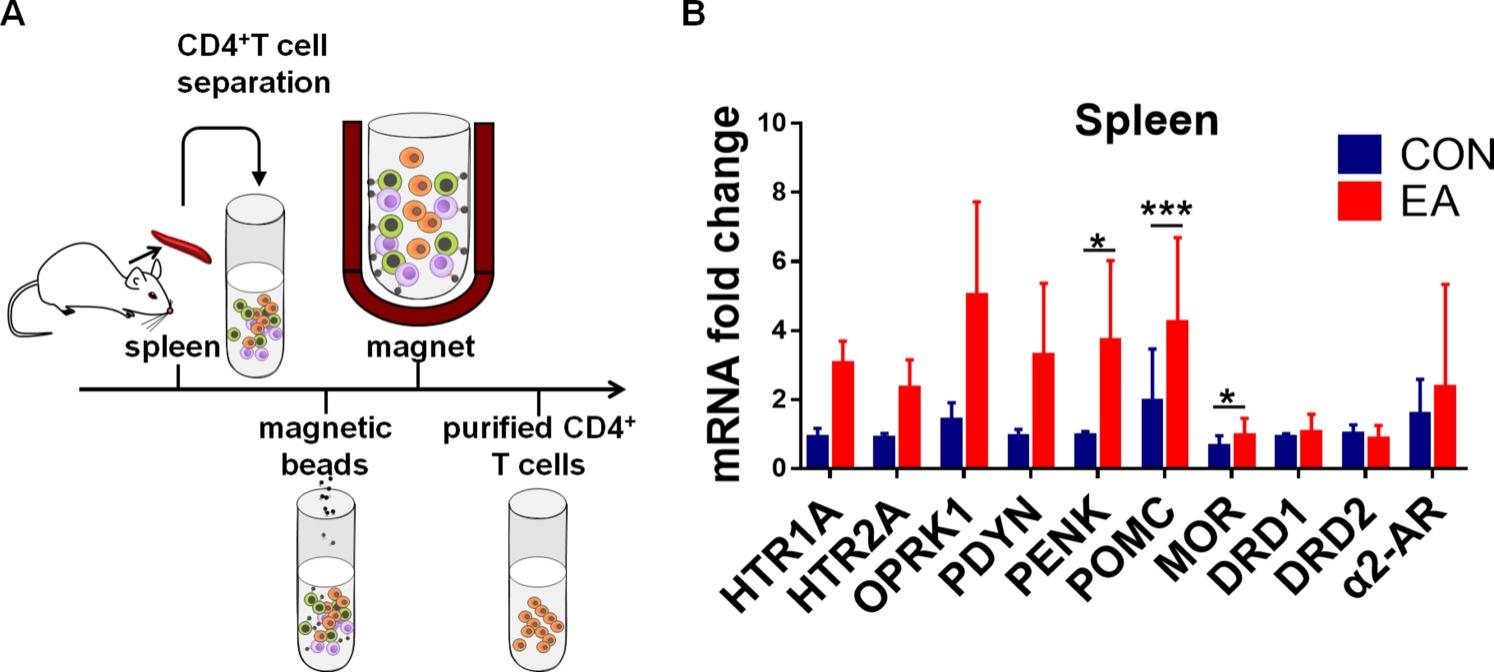


**Supplementary Figure S4**

**A** CD4^+^ T cells in rat spleen were sorted with a magnet using the MojoSort Rat Isolation Kit, and the purity was confirmed by flow cytometry. B Gene expression of analgesic protein in spleen from both control and EA group was detected by RT-qPCR. n=5. Data are shown as mean ± SD, **p*<0.05, ****p*<0.001.


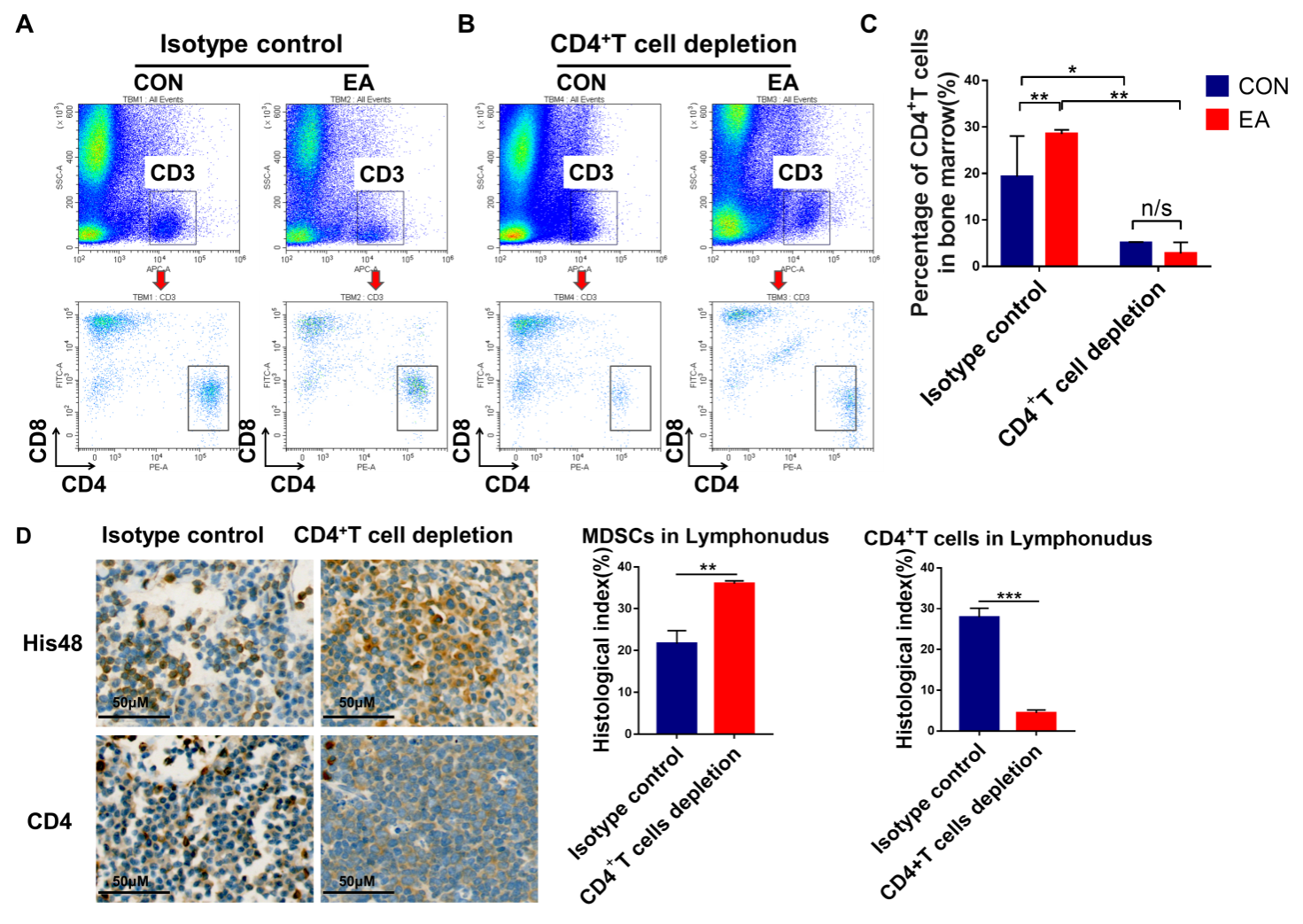


**Supplementary Figure S5**

**A-C** Anti-CD4^+^ T cell antibody was applied to deplete CD4^+^ T cells followed by EA treatment; and the effect in bone marrow was detected by flow cytometry, n=3.Data are shown as mean ± SD, **p*<0.05, ***p*<0.01.**D** Immunohistochemistry was used to detect MDSCs and CD4^+^ T cells in the lymph nodes of rats after CD4^+^ T cells depletion. The results were observed under a microscope (×400). Data are shown as mean ± SD, n=3, ***p*<0.01, ****p*<0.001.


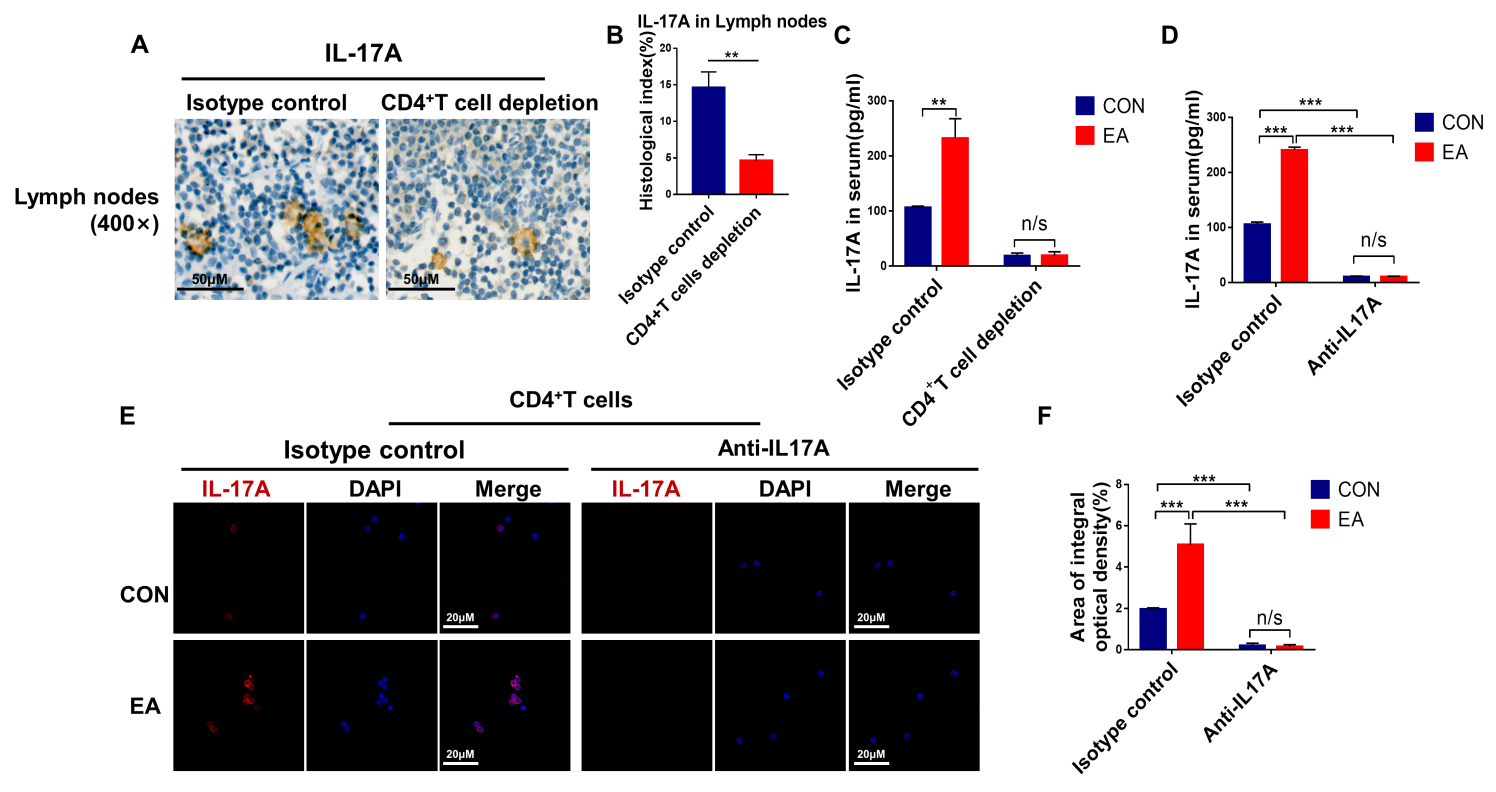


**Supplementary Figure S6**

**A-B** Immunohistochemistry was used to detect the expression of IL17A in lymph nodes of BPRA rats after EA treatment with CD4^+^ T cells depletion, the results were observed under a microscope (×400). Data are shown as mean ± SD, n=3, ***p*<0.01, ****p*<0.001. **C** Concentration of IL17A in serum of BPRA rats with CD4^+^T cells depletion was detected using ELISA kit after EA treatment, Data are shown as mean ± SD, n=7, ***p*<0.01. **D** Rats were treated with 10mg/kg anti-IL17A antibody, or control mouse IgG by intra-peritoneal injections once a week for two months to neutralize IL17A before EA treatment. Concentration of IL17A in serum was detected using ELISA kit. n=3.Data are shown as mean ± SD, ****p*<0.001. **E-F** CD4^+^ T cells sorted from spleen were stimulated with CD3, CD28 antibodies and Brefeldin A solution. Immunofluorescence was used to detect the expression of IL17A in rat spleen CD4^+^ T cells after IL17A depleted, n=3. Data are shown as mean ± SD, ****p*<0.001.
